# Book review: *An Introduction to Primate Conservation*

**DOI:** 10.5194/pb-4-69-2017

**Published:** 2017-03-29

**Authors:** Eckhard W. Heymann

**Affiliations:** Verhaltensökologie & Soziobiologie, Deutsches Primatenzentrum, Leibniz-Institut für Primatenforschung, Göttingen, Germany

*Wich, S. A. and Marshall, A. J.: An Introduction to Primate Conservation, Oxford University Press, 2016, xvi + 302 pp., ISBN 978-0-19-870338-9, EUR 92.40 (hardcover), ISBN 978-0-19-870339-6, EUR 39.95 (paperback), 2016.*

“Saving the Lion Tamarin” was the programmatic title of a book by Bridgwater (1972), perhaps the first book explicitly dedicated to primate conservation. The activities that followed the publication of this book created a success story in primate conservation: although still threatened, the golden lion tamarin is now in a much better situation than it was in the early 1970s (Kleiman and Rylands, 2002). Many other books dealing with primate conservation have followed since then, either focussing on specific taxa (e.g. Kleiman, 1977), specific geographic regions (e.g. Nadler et al., 2010), and specific threats or problems (e.g. Marsh and Chapman, 2013) or covering different taxa and conservation issues (e.g. Prince Rainier and Bourne, 1977; Marsh and Mittermeier 1986). The first comprehensive synthesis of the many aspects and problems in primate conservation was undertaken by Cowlishaw and Dunbar (2000). Since then, conceptual and methodological advances have been made, so a book presenting these advances and at the same time reviewing the state of the art of “classical” conservation issues was overdue. Thus, the book by Wich and Marshall is timely.

It is an edited book in which specialists for different topics and taxa review recent advances. The book comprises in total 18 chapters, including a general introduction to primate conservation and an outlook (chapters 1 and 18 by the editors) that enframe the other, more specific, chapters. Several of the other chapters deal with “classical” topics like habitat destruction (Chapter 7 by Irwin), hunting (Chapter 9 by Fa and Tagg), and trade (Chapter 8 by Nijman and Healy), while others present more recent or novel aspects like the impact of infectious diseases (Chapter 10 by Nunn and Gillespie) and of climate change (Chapter 11 by Korstjens and Hillyer). Two chapters are strongly method oriented: that on conservation genetics, including a genomic perspective (Chapter 5 by Lynn and colleagues), and that concerning concepts and methods for estimating primate abundance and distribution (Chapter 6 by Campbell and colleagues). A chapter that I especially liked is “Why conserve primates?” by the editors. It provides a thorough and balanced discussion which confronts the “primatocentric” perspective often taken by primatologists with the reality and the problems such a perspective may cause. What I also liked is the combination of reviews with case studies, which provides a lively reading. Not explicitly mentioning the remaining chapters does not mean they were less important or good – all chapters and the book as a whole are important. The book fills a gap and will do a great job in training the next generation of primatologists and conservationists in understanding the proximate causes and mechanisms of the primate extinction crisis. It will also be very useful to professionals for updating their knowledge in “classical” fields of primate conservation biology and learning about new approaches like the REDD+ initiative. Therefore, I sincerely recommend it to everyone interested in this topic.

The only thing that I missed from the book (but also from all previous ones on primate conservation) is a chapter that deals with the ultimate causes of threats to primates, which are essentially of political and economic nature. We scientists are often too reluctant to make political statements and to name the real causes of the primate extinction crisis: the exploitation of nature and human labour in primate habitat countries by industrialized countries and the increasing discrepancy in the distribution of wealth between industrialized and habitat countries, or – more succinctly – the profit- and competition-oriented economy.

Despite numerous books and articles on primate conservation and many conservation initiatives and activities, the situation of primates has been aggravated: more primate species are threatened than ever before and – on a global scale – in first place by habitat destruction (Estrada et al., 2017), which was already identified as a major threat decades ago (Myers, 1986). On a sceptical if not cynical note, one may ask what all the books and articles dealing with primate conservation have achieved and whether it worth printing more books and articles on primate conservation. Despite this scepticism, I think it is still worthwhile and of utmost importance to fight for the continued existence of our biological relatives. The book by Wich and Marshall will contribute to this.
